# Incidence and etiology of hospitalized acute respiratory infections in the Egyptian Delta

**DOI:** 10.1111/irv.12409

**Published:** 2016-08-12

**Authors:** Emily Rowlinson, Erica Dueger, Adel Mansour, Nahed Azzazy, Hoda Mansour, Lisa Peters, Summer Rosenstock, Sarah Hamid, Mayar M. Said, Mohamed Geneidy, Monier Abd Allah, Amr Kandeel

**Affiliations:** ^1^ Global Disease Detection and Response ProgramUS Naval Medical Research Unit No. 3CairoEgypt; ^2^ US Centers for Disease Control & PreventionAtlantaGAUSA; ^3^ US Naval Medical Research Unit No. 3CairoEgypt; ^4^ Preventive SectorMinistry of Health and PopulationCairoEgypt

**Keywords:** acute respiratory infections, hospitalization, influenza, population surveillance, respiratory syncytial viruses, viral respiratory pathogens

## Abstract

**Introduction:**

Acute Respiratory Infections (ARI) are responsible for nearly two million childhood deaths worldwide. A limited number of studies have been published on the epidemiology of viral respiratory pathogens in Egypt.

**Methods:**

A total of 6113 hospitalized patients >1 month of age with suspected ARI were enrolled between June 23, 2009 and December 31, 2013. Naso‐ and oropharyngeal specimens were collected and tested for influenza A and B, respiratory syncytial virus, human metapneumovirus, adenovirus, and parainfluenza viruses 1–3. Blood specimens from children 1–11 months were cultured and bacterial growth was identified by polymerase chain reaction. Results from a healthcare utilization survey on the proportion of persons seeking care for ARI was used to calculate adjusted ARI incidence rates in the surveillance population.

**Results:**

The proportion of patients with a viral pathogen detected decreased with age from 67% in patients age 1–11 months to 19% in patients ≥65 years of age. Influenza was the dominant viral pathogen detected in patients ≥1 year of age (13.9%). The highest incidence rates for hospitalized ARI were observed in children 1–11 months (1757.9–5537.5/100 000 population) and RSV was the most commonly detected pathogen in this age group.

**Conclusion:**

In this study population, influenza is the largest viral contributor to hospitalized ARIs and children 1–11 months of age experience a high rate of ARI hospitalizations. This study highlights a need for surveillance of additional viral pathogens and alternative detection methods for bacterial pathogens, which may reveal a substantial proportion of as yet unidentified etiologies in adults.

## 1 Introduction

Acute respiratory infections (ARIs) are a globally significant cause of mortality and morbidity. Lower respiratory infections are the third leading cause of death worldwide^1^ and nearly two million childhood deaths worldwide are attributable to ARI, with the vast majority occurring in developing countries.^2^ Prevention and adequate treatment of ARIs can have a significant impact on population morbidity and mortality. However, there is a dearth of data on the proportional contribution of distinct etiologies to the overall burden and locally significant causes of ARIs, especially in resource‐limited settings.

The World Health Organization estimates that in 2013, over 8% of all deaths in the Eastern Mediterranean Region were attributable to ARIs.^1^ However, regional data on morbidity and etiology of these infections are lacking. Certain risk factors for severe respiratory disease are widely prevalent in Egypt, including diabetes (9.3%)^3^ and liver disease (specifically hepatitis C, 14.7%).^4^, ^5^ Additionally, nearly 20% of adult Egyptians and 38% of adult males regularly use tobacco products.^6^ Data on the burden and etiologies of ARI are especially pertinent for Egypt, a nation with a high birth rate and 11% of its population consisting of children <5 years of age^7^ and other countries in the region having similar age structures. A limited number of studies have been published on the epidemiology of viral respiratory pathogens in Egypt,^8^, ^9^, ^10^, ^11^ with the majority focused on one clinical site and a time frame of less than two years.

The United States Centers for Disease Control and Prevention's (CDC) International Emerging Infections Program (IEIP), in collaboration with local host governments, has conducted population‐based surveillance for pathogens of public health importance in several countries since 2004. Sites in Kenya, Thailand, Egypt, Guatemala, China, South Africa, and Bangladesh currently conduct or have conducted surveillance for viral and bacterial pathogens associated with ARI. Surveillance over multiple years provides data on the epidemiology and proportional contribution of each pathogen to overall acute respiratory disease burden at each site. The IEIP site in Damanhour, Egypt, is a collaboration between the CDC, Egypt's Ministry of Health, and the United States Naval Medical Research Unit No. 3 (NAMRU‐3) to conduct population‐based surveillance for ARI, acute febrile infections (AFI), and acute infectious neurological disease (AIND). Population‐based surveillance provides a more accurate picture of disease incidence than sentinel surveillance because it allows for adjustments to estimates based on local patterns of healthcare utilization.^12^

Damanhour is a mixed urban/rural area in Egypt's Nile Delta Region. At the time of the last census in 2006, Damanhour district's population was estimated at 685 641, with 65% of the population residing in rural villages. Population demographics in Damanhour mirror national trends, with 50% of the population under the age of 20 [CAPMAS]. Egypt's crude birth rate of 12/1000 population [World Bank] is indicative of a continued youth bulge in the coming years. Virtually all residents of Damanhour are of Egyptian ethnicity. No estimates of influenza vaccine coverage in Egypt have been published to date, although it is available in the private sector. Pneumococcal vaccine is not included in Egypt's immunization schedule,^13^ and *Haemophilus influenzae b* (*Hib*) vaccine was added to the immunization schedule in February 2014 (personal communication with Dr. Amr Kandeel, Ministry of Health).

This manuscript presents the findings of the first three and a half years of population‐based ARI surveillance from IEIP Egypt's site in Damanhour.

## 2 Methods

### 2.1 Study population

Population‐based surveillance for ARI was initiated in Damanhour district in June 2009.

Surveillance was initiated in three government referral hospitals based on the results of a 2008 Healthcare Utilization Survey (HUS).^9^ Patients admitted to these hospitals are referred by local outpatient facilities. This survey was repeated in 2011/2012, and the combined results indicated that >95% of persons hospitalized for ARI in Damanhour were admitted to government facilities. Study staff reviewed hospital admission logbooks six days per week to identify patients admitted for suspected ARI. Identified patients admitted during the current day or the previous night were screened for eligibility daily during rounds. Patients admitted on Fridays, the only non‐working day for governmental facilities in Egypt, or on holidays, were screened the following morning for eligibility. Patients were eligible if they met a broad set of symptomologies indicative of severe respiratory infection (Table 1). Patients aged 1 month to 5 years were eligible if they met either adult or pediatric enrollment criteria. Patients were excluded if they were <31 days of age, not a resident of Damanhour district, or had already been enrolled in the surveillance system for the current episode of illness. Patients <31 days of age were excluded because physicians were not comfortable performing nasopharyngeal swabs on ill patients in this age group. After consent was obtained from the patient or their legal guardian, study personnel conducted patient interviews and chart reviews to complete standardized data collection forms detailing demographic variables, relevant medical history, and symptomology of the patients’ current illness. Oxygen saturation was measured at enrollment using a finger pulse oximeter. Chest X‐rays were performed when clinically indicated by the attending physician, and any interpretations recorded by either the attending physician or pulmonologist were abstracted from the patients’ medical record.

**Table 1:** Case definitions used for acute respiratory infection enrollment

All patients ≥31 d	Patients ≥31 d to <5 y of age only^a^
Hospitalized with moderate to severe acute lower respiratory tract illness consisting of:One of the following signs of acute infection Fever ≥38°C History of fever with this illness Current hypothermia less than 35.5°C Abnormal white blood cell count or differential andOne of the following sings/symptoms: Cough Sore through Abnormal breath sounds Tachypnea Sputum production Hemoptysis Chest pain Dyspnea	Hospitalized and meeting one of the two following case definitions:1. Integrated Management of Childhood Illness—Severe PneumoniaTachypnea defined as: Respiratory rate>50/min for infants ≥31 d to <1 y Respiratory rate>40/min for children 1 to <5 y andOne of the following: Unable to drink or breastfeed Lethargic or unconscious Vomits everything Convulsions Nasal flaring Grunting Oxygen saturation<90% Chest indrawing Stridor in a calm child 2. Integrated Management of Childhood Illness – Moderate PneumoniaTachypnea defined as Respiratory rate>50/min for infants ≥31 d to <1 y Respiratory rate>40/min for children aged 1 to <5 y and Cough or Sore throat

a Patients aged ≥31 d to 5 y were eligible if they met either adult or pediatric enrollment criteria.

Nasopharyngeal (NP) and oropharyngeal (OP) specimens were collected using a dry polyester‐tipped swab (Fisher Scientific, Pittsburgh, PA, USA) from enrolled hospitalized patients, placed in 2 mL of liquid viral transport media, and stored at 4°C in hospital laboratories for a maximum of two days. Specimens were tested at the International Emerging Infections Program (IEIP) laboratory in Damanhour, and an aliquot of each specimen was stored in liquid nitrogen and shipped weekly to the U.S. Naval Medical Research Unit No. 3 (NAMRU‐3) reference laboratory in Cairo, Egypt, for confirmation. All specimens were tested for the presence of ribonuclease P (RNP) gene, an internal positive control used as an indicator of sufficient human cellular material for viral pathogen testing. Specimens were tested by real‐time reverse‐transcription PCR (rRT‐PCR) for a panel of viral respiratory pathogens, including influenza A and B, respiratory syncytial virus (RSV), human metapneumovirus (hMPV), adenovirus, and parainfluenza viruses (PIV) 1–3, using CDC‐developed assays and testing protocol.^14^, ^15^, ^16^, ^17^

Blood specimens were collected from enrolled children ≥31 days to <5 years of age by drawing 3–5 mL of blood directly into BACTEC™ blood culture bottles. Blood culture bottles were stored at room temperature in hospital laboratories, transferred to the IEIP Damanhour laboratory daily for incubation in the BACTEC™ blood culture system at 35–36°C, and observed daily for signs of microbial growth. Specimens with growth were examined by gram stain, and cultured onto chocolate, blood, and MacConkey agars for further identification. After five days of incubation in the IEIP Egypt Damanhour laboratory, 1 mL aliquots from the BACTEC™ blood culture bottles were transferred into a cryovial and sent in a liquid nitrogen tank to NAMRU‐3 for PCR analysis for *Streptococcus pneumonia*.

### 2.2 Data management and statistical analyses

Data collected on standardized forms were reviewed by clinical supervisors and senior‐level investigators for consistency and errors and double‐entered using Microsoft Access. Statistical analyses were conducted using SPSS version 16.0.^18^ Patients older than ≥31 days screened for ARI between June 1, 2009, and December 31, 2012, were included for analysis. Incidence rates for ARI were calculated using methods described previously.^9^ Numerators were the total number of patients eligible for ARI enrollment, stratified by sex, residence (rural vs urban), and age group (1–11 months, 1–4, 5–19, 20–49, 50–64, and ≥65 years). Age groups were based on recommendations from the WHO^19^ and modified due to availability of population data age groupings from CAPMAS. Gender data were not collected from eligible patients who did not enroll; these patients were assumed to be proportionally equal in gender to enrolled patients stratified by age group and residence (rural vs urban). Unadjusted age‐specific hospitalized ARI rates were calculated by dividing the number of enrolled ARI patients in each age category by the corresponding age‐specific population denominator. Rates were adjusted for patients eligible but not enrolled, stratified by sex, age, and residence (rural vs urban). No adjustment for healthcare utilization was necessary due to the near universal use of surveillance facilities for hospitalization reported by healthcare utilization survey respondents. ARI hospitalization rates were calculated for all years separately and combined, and for rural and urban locations separately. Urban and rural rates of ARI were age‐adjusted to the total Damanhour population demographics.

### 2.3 Ethics statement

This study was approved by scientific and institutional review boards (IRBs) (protocol #906) at NAMRU‐3 in Cairo, Egypt, and the IRB (protocol #5641) at the Centers for Disease Control and Prevention in Atlanta, GA, USA. The protocol also received approval from the Egyptian Ministry of Health and Population in Cairo. A waiver of documentation of consent from patients/guardians was obtained from both IRBs. Surveillance staff verbally described all study procedures to the patients and provided them with a written document detailing all procedures; study staff then obtained verbal consent from patients over 17 years of age, verbal assent, and parental consent for patients 5–17 years of age, and parental/guardian consent for patients <5 years of age. Study staff signed consent forms documenting verbal consent/assent from patients/guardians, and a record of completed consent procedures was maintained for all patients.

## 3 Results

### 3.1 Enrollment and demographics

Between June 23, 2009, and December 31, 2013, a total of 11 929 patients with suspected moderate or severe ARI were screened for enrollment. Of these patients, 6684 (56%) were determined to be eligible by symptomology, residency, and age requirements and 6113 (91.5%) were enrolled. Over one‐third of all enrolled ARI patients were 1 month to <5 years of age, and nearly three quarters were rural area residents (Table 2). A nearly equal proportion of males and females were enrolled, although males and rural residents comprised a slightly higher percentage of enrolled children aged 1 month to <5 years (58%). Of the years where a full calendar year of surveillance was conducted (2010–2012), the months with the highest percentage of patients eligible for enrollment were October and December (10.3% and 11.4%, respectively). Less than 1% of our study population reported receiving influenza vaccination in the past 12 months, and no patients reported receiving pneumococcal or *Haemophilus influenzae b* (*Hib*) vaccine.

**Table 2:** Characteristics of patients hospitalized with acute respiratory infection

	Urban, N (%)	Rural, N (%)	Total, N (%)
Total	1590 (26)	4523 (74)	6113 (100)
Demographics			
Age (y)			
<1	218 (14)	831 (18)	1047 (17)
1–4	252 (16)	940 (21)	1185 (19)
5–19	165 (10)	508 (11)	671 (11)
20–49	438 (28)	1195 (26)	1607 (26)
50–64	347 (22)	766 (17)	1093 (18)
65+	170 (11)	283 (6)	446 (9)
Pregnant women^a^	10 (1)	55 (3)	65 (7)
Signs and symptoms			
Symptom onset ≤7 d	1242 (78)	3481 (77)	4723 (77)
History of fever (with this illness)	1558 (98)	4468 (99)	6026 (99)
Cough	1541 (97)	4401 (97)	5942 (97)
Current fever ≥38°C	683 (43)	2357 (52)	3040 (50)
Tachypnea	805 (51)	2008 (44)	2813 (46)
Dyspnea	917 (58)	1946 (43)	2863 (47)
Abnormal breathing sounds	1226 (77)	3284 (73)	4510 (74)
Sputum production	1118 (70)	2791 (62)	3909 (50)
Hemoptysis	54 (3)	110 (2)	164 (3)
Clinical findings			
Antibiotics prior to admission	959 (60)	2855 (64)	3814 (62)
Reported comorbid chronic condition	421 (26)	759 (17)	1180 (19)
Intensive care unit^b^	88 (6)	159 (4)	147 (2)
Ventilated	15 (1)	8 (<1)	22 (15)
Oxygen saturation performed	999 (62)	2790 (62)	3765 (62)
Oxygen saturation <90	106 (7)	258 (6)	364 (10)
Chest X‐ray performed	947 (60)	2330 (52)	3277 (54)
Result abnormal	396 (42)	891 (38)	1287 (39)
Infiltrates	140 (15)	265 (11)	405 (32)
Pleural effusion	23 (2)	45 (2)	68 (5)
Consolidation	204 (22)	537 (23)	741 (58)
Cavitation	1 (<1)	9 (<1)	10 (<1)
Death in hospital	23 (1)	27 (<1)	50 (<1)
Mean length of hospitalization (d)	6.7	5.2	5.6

a As a percentage of women age 15–49.

b Sixty‐five patients missing Ventilation, ICU, and CXR information.

### 3.2 Signs, symptoms, and clinical characteristics of enrolled patients

Nearly all enrolled ARI patients presented with cough (97%) and history of fever with the current illness (98%) (Table 2). Less than half of these patients had evidence of current fever ≥38°C, tachypnea, dyspnea, or sputum production. Nearly three quarters of patients had abnormal breathing sounds and approximately one‐quarter reported symptom onset ≥7 days prior to enrollment. Almost 20% of patients reported a comorbid chronic condition. The proportion of patients reporting a chronic condition increased with age, with 29% of patients age 20–49, 41% of patients age 50–64, and 43% of patients over age 65 reporting ≥1 chronic condition. Among these patients with a chronic condition, hypertension, diabetes (each 31%), liver disease (including hepatitis C) (16%), and asthma (12%) were most commonly reported. These chronic conditions were reported exclusively in patients >20 years of age. In children aged 1 month–11 years reporting a chronic condition, the most commonly reported chronic condition was congenital heart defects (24 patients, 53%), followed by asthma (5, 11%). Among older children and adolescents reporting a chronic condition, 33% of patients aged 1–4 years and 32% of patients aged 5–19 years reported asthma.

A small percentage (2.4%) of patients were admitted to the ICU during hospitalization (Table 2), and even fewer were placed on mechanical ventilation. Nearly 10% of the patients with an oxygen saturation reading had levels <90%. About half of enrolled patients had a chest X‐ray performed. A higher percentage of adults 50–64 (64%) and ≥65 (63%) years of age had a chest X‐ray performed. Age groups with the highest percentage of abnormal chest X‐rays were adults aged ≥65 years (52%), adults aged 50–64 years (47%), and infants aged 1–11 months (41%). Of those with abnormal chest X‐ray results, the most common finding was pulmonary consolidation, followed by infiltrates (Table 2). Nearly half of the 50 patients who died in hospital were ≥65 years of age. Patients who died in hospital were significantly more likely to report a chronic condition one or more of the following comorbidities: liver disease, hypertension, asthma, and diabetes (60% vs 19%, *P*<.01). The case fatality rates for age groups were as follows: 1–11 months, 0.4%; 1–4 years, 0.3%; 5–19 years, 0%; 20–49 years, 0.4%; 50–64 years, 1.1%; ≥65 years, 2.6%. Cause of death was not recorded for this study. Of the 50 patients who died in hospital, 24 had a chest X‐ray performed prior to death. Twenty‐two (92%) of these chest X‐rays were deemed abnormal by the reviewing radiologist; 11 noted pleural consolidation and 10 noted pleural infiltrates. A viral etiology was identified in only 14% of the patients who died during hospitalization. Three of the patients who died during hospitalization tested positive for influenza A only (1 each pandemic A(H1N1)2009, H1N1, and H3N32) and 1 each tested positive for RSV and adenovirus. Of the eight patients aged 1 month to <5 years who died during hospitalization, two (25%) had viral co‐infections identified (PIV‐3 and hMPV; PIV‐2 and adenovirus) compared to 6% of children who were discharged (*P*=.04).

There were some differences between rural and urban patients with respect to symptomology. A lower proportion of rural patients had dyspnea, tachypnea, abnormal breathing sounds, and sputum production at admission (Table 2). Additionally, a lower proportion of rural patients reported a chronic condition. The mean length of hospitalization was shorter for rural patients compared with urban patients (5.2 vs 6.7 days).

### 3.3 Diagnostic results

**Figure 1:**
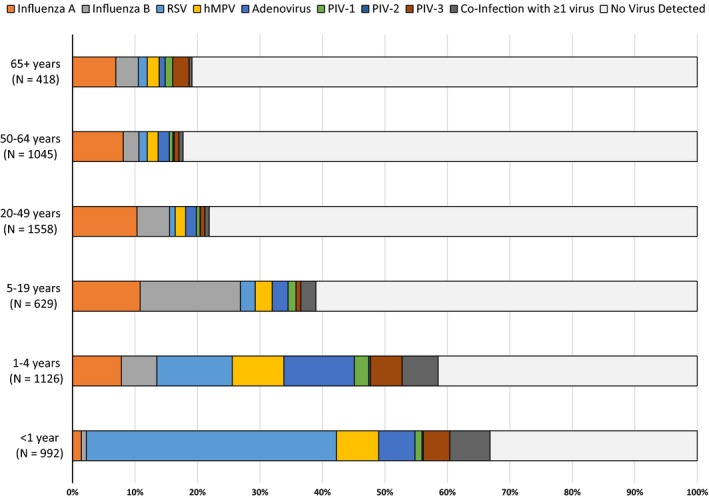
Viral pathogens identified in NP/OP swabs by RT‐PCR by age group

Results of the rRT‐PCR viral pathogen panel on NP/OP specimens are presented in Table 3. At least one viral pathogen was detected in 36% of specimens collected. Pathogen detection was highest in patients whose samples were taken 3–5 days after onset of symptoms (42%). Pathogen detection was 32% in patients presenting 11–14 days after symptom onset and decreased to 24% in patients presenting >2 weeks after symptom onset. Overall, the most commonly detected pathogen was influenza, followed by RSV, adenovirus, and hMPV. The profile of pathogens detected in different age groups is presented in Fig. 1. The largest proportion of RSV‐positive patients occurred among children aged 1–11 months, while influenza was the dominant viral pathogen detected among patients >5 years of age. Both hMPV and adenovirus were most commonly detected in patients aged 1 month to <5 years. Influenza A was the dominant influenza virus detected in all age groups, except in patients aged 5–19 years. The proportion of patients with a viral pathogen detected decreased with age, from 67% in patients aged 1–11 months to 19% in patients aged ≥65 years. Co‐infection with ≥1 viral pathogen was most common in patients aged 1 month to <5 years (82% of 153 viral co‐infections); the most commonly detected co‐infection was adenovirus and RSV (31.7% of 153 viral co‐infections detected). Adenovirus was the pathogen most commonly identified among patients with a viral co‐infection (96, 68.0%).

**Table 3:** Viral Pathogens Detected in NP/OP Swabs and Bacterial Pathogens Detected in Blood Specimens

	N	%
NP/OP swab collected	5768	94.4
Viral pathogens^a^		
Influenza^b^	799	13.9
Influenza A	487	8.4
Influenza B	316	5.5
Respiratory syncytial virus	669	11.6
Adenovirus	355	6.2
Human metapneumovirus	266	4.6
Parainfluenza virus 1	73	1.3
Parainfluenza virus 2	9	0.2
Parainfluenza virus 3	155	2.7
Co‐Infection with 2 viruses	149	2.4
Co‐Infection with 3 viruses	4	0.1
Bacterial pathogens		
Blood specimens collected^c^	2150	96.3
Antibiotic use prior to specimen collection	2034	91.1
Coagulase‐negative Staphylococci	354	16.5
*Escherichia coli*	2	<0.1
Gram‐positive rods	40	1.9
*Klebsiella pneumoniae*	3	0.1
*Psuedomonas aeruginosa*	5	0.2
*Staphlyococcus aureus*	2	<0.1
Brucella	1	<0.1
*Salmonella* typhi	1	<0.1
Viridans streptococci	5	0.2

a Includes detected co‐infections.

b Four patients co‐infected with influenza A and B.

c As a percent of 2232 enrolled children aged <5 y.

Blood culture was performed on 96% of enrolled ARI patients aged 1 month to <5 years. The vast majority (94.1%) of these patients had documented antibiotic use prior to either hospitalization or specimen collection. Pathogenic bacteria were detected in only a small percentage of blood specimens (Table 3).

### 3.4 Seasonality of pathogens

**Figure 2:**
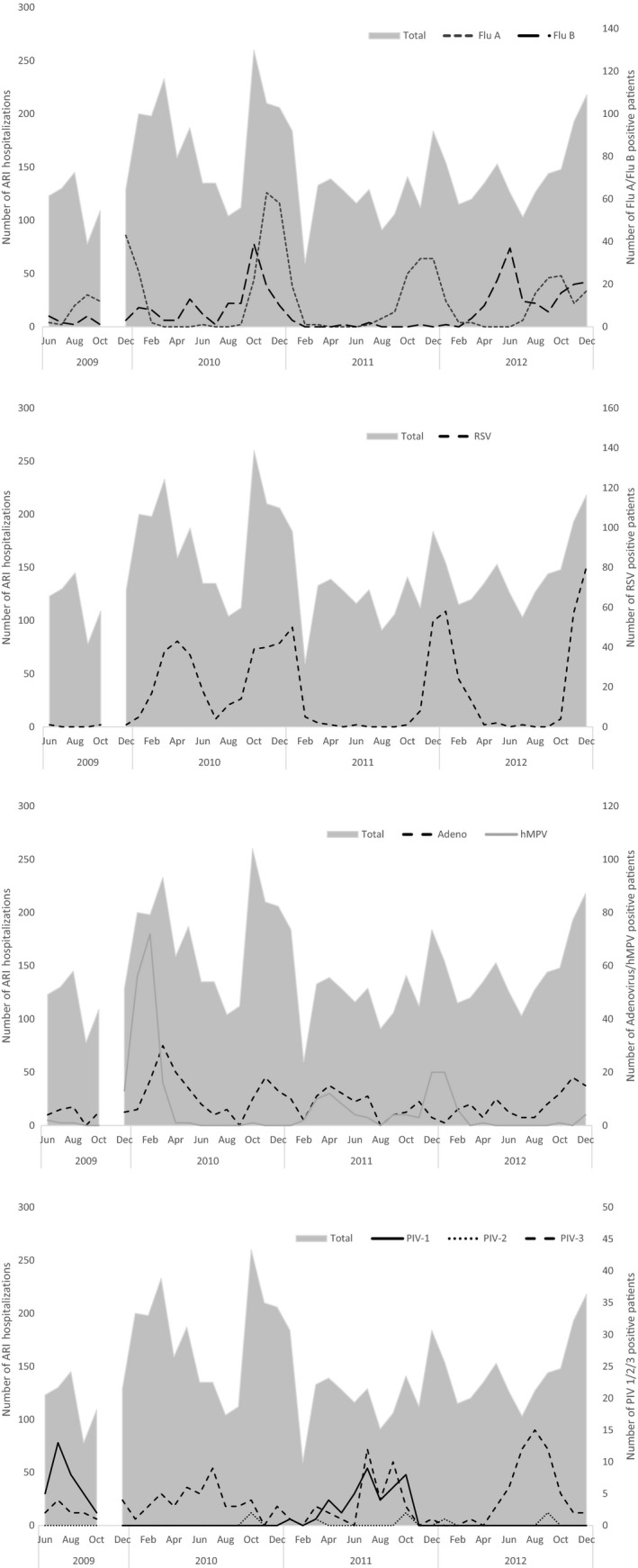
Seasonality of respiratory pathogens

The seasonality of viral pathogens is shown in Fig. 2. Influenza A demonstrated consistent seasonality initiating in August/September and peaking on or shortly before December; influenza B demonstrated an irregular seasonality. Adenovirus persisted at low levels throughout the data collection period. A marked peak in detected hMPV cases occurred from December to March of 2009/2010 and was not replicated in subsequent years. RSV demonstrated consistent November–February seasonality in the number of cases, with the exception of 2009 when two distinct peaks occurred in April and again in October–January. PIV‐1 and PIV‐2 did not seem to follow a specific seasonality, while PIV‐3 circulation regularly peaked between June and September.

The incidence of hospitalized ARI varied markedly between years of data collection, age groups, and geographic location. The overall incidence ranged from a high of 315.9 cases per 100 000 population in 2010 to a low of 219.6 per 100 000 population in 2011. The highest incidence rates for hospitalized ARI were consistently observed in children aged 1–11 months (Table 4), which ranged from 1758 to 5538/100 000 population during 2009–2012. Incidence rates for hospitalized ARI were comparable between patients aged 1–4 years and those aged ≥65 years. The age‐adjusted rate of hospitalized ARI in the rural population was consistently higher than that of the urban population.

**Table 4:** Observed incidence of ARI by age group

All ages	2009^a^		2010		2011		2012		Overall	
	N	Incidence per 100 000	N	Incidence per 100 000	N	Incidence per 100 000	N	Incidence per 100 000	N	Incidence per 100 000
Unadjusted	1141	265	2179	295.3	1459	194.7	1685	212.2	6049	265.2
Adjusted		274.6		315.9		219.6		234.2		289.6
Age group – adjusted										
1–11 mo	71	1757.9	384	5537.5	252	3956.6	340	4929.5	1047	5134.9
1–4 y	129	362.0	446	753.4	278	516.1	332	575.2	1185	681.2
5–19 y	67	52.2	332	153.0	190	87.1	174	75.7	671	101.1
20–49 y	208	112.3	547	173.4	401	128.3	452	135.4	1607	166.4
50–64 y	161	420.9	344	488.4	410	545.5	297	374.6	1093	491.2
65+ y	72	543.3	126	582.4	113	488.0	126	488.8	446	632.8
Residence – adjusted										
Rural	497	202.1	1581	371.2	1133	278.5	1248	278.5	2048	346.2
Urban	340	129.6	531	199.5	375	135.2	437	169.8	1590	192.5

a June 23, 2009–December 31, 2013.

## 4 Discussion

Hospitalization of patients for moderate or severe acute respiratory infection remains an important and complex public health concern in the Egyptian Delta. The incidence of patients hospitalized for moderate or severe ARI during our study period was 290/100 000 population, with higher rates in very young children aged 1–11 months (5135/100 000 population overall for the period 2009–2012). Our incidence estimates of hospitalized ARI were comparable to previously published data from several similar studies in Asia and Central America. The very high estimated incidence rate for children aged 1–11 months in our study population (5135/100 000) is similar to estimates from Thailand (5772/100 000).^20^ The incidence for all age groups combined was similar to published estimates from Guatemala (128/100 000),^21^ although our age‐specific estimates were much higher. This may be explained by differences in healthcare utilization and hospitalization rates between the two populations. Published incidence rates from a population‐based surveillance site in Kenya were much higher than our estimates (699.8/100 000),^22^ but did not include fever as enrollment criteria, likely increasing their overall capture of respiratory disease due to non‐infectious etiologies. Compared with the above studies, very few ARI deaths occurred during hospitalization in our study population, and an even smaller proportion of these deaths were associated with an identified viral pathogen. Seven percent of enrolled ARI patients either left against medical advice or were transferred to another hospital; therefore, we have no information on their outcome after they left the study hospitals. Transferred cases may have been more severe and in need of specialized care, and it is possible that rates of death may have been higher in these patients than those who remained hospitalized in the study hospitals.

Peaks of PIV‐3, influenza A, and RSV were well defined and the majority of disease burden occurred during these peaks, with the dominant etiology varying between years of our study period. The early peak of RSV in April 2009 may reflect a delay in the previous year's winter peak, as there was no evidence of increased RSV positivity in fall 2008 as was seen in subsequent years. While all pathogens in the diagnostic panel were identified across age groups, only influenza was found more commonly in adults compared to children. Adenovirus, hMPV, and PIV‐1, PIV‐3 were documented as important contributors to the burden of hospitalized ARI in young children, especially in children aged 1–4 years; however, they were seen relatively infrequently in older age groups. This is consistent with a wide body of published data on viral etiologies in young children.^10^, ^23^, ^24^, ^25^

High incidence of RSV infections has been reported previously in this population,^9^ and this study confirms RSV as the predominant cause of viral ARI, and likely all ARI, in children aged 1–11 months. RSV was identified in nearly 40% of all children aged 1–11 months hospitalized with ARI. Our data demonstrate that RSV causes a much higher burden of hospitalized ARI in children compared with influenza, similar to studies from other countries.^9^, ^14^, ^20^, ^26^ This study does not capture the high burden of RSV in children who may not require hospitalization, but experience substantial morbidity. Additionally, children <31 days were not included in this study, but have been shown to have high rates of RSV infection.^27^, ^28^ With the majority of ARI hospitalizations occurring in this age group, and the disproportionate number of these hospitalizations due to RSV, the burden of RSV in this population cannot be understated.

In this study population, influenza is the largest viral contributor to hospitalized ARIs. This is inconsistent with similar surveillance data from Guatemala in the same time period, during which influenza was identified in a much smaller proportion of patients compared with RSV and adenovirus.^21^ In that study, however, over 40% of enrolled patients were under 5 years of age, and consistent with our data, RSV was the most common etiology in that age group. Relatively few children under 5 years of age had influenza infection compared with older age groups; influenza A and B comprised nearly half of the identified etiologies in adults over the age of 65, and well over half in younger adults. The high rate of influenza detection in adolescents and younger adults may be due to circulation of pandemic A(H1N1)2009 during the study period, which has been shown to disproportionately affect this age group.^29^ Given the virtual absence of individuals vaccinated for influenza in this study population, vaccination of high‐risk populations has the potential to avert a proportion of hospitalized ARIs in Egypt. Co‐infections with ≥1 virus were found in approximately 3% of hospitalized ARI patients. Over a quarter of all adenovirus infections identified occurred as a co‐infection with at least one other virus. The fact that the vast majority of co‐infections were detected in children and the relative infrequency of adenovirus detection in adults may partially explain this. Co‐infection with adenovirus was also frequently identified in a separate study of Egyptian children under 5 years of age.^10^ In this population, children aged 1 month to <5 years who died during hospitalization were significantly more likely to have had a viral co‐infection than those who were discharged. Although previously published data suggest viral co‐infection can be associated with more severe illness and poor outcomes,^30^ a review of published studies on the subject revealed conflicting conclusions on the association between viral co‐infection and disease severity in hospitalized children.^31^ Regardless, it is important to note that the pathogens of interest in this study may have some degree of baseline carriage or circulation in healthy populations^32^, ^33^, ^34^; therefore, it is not possible to conclusively ascribe causality to these pathogens in all patients with positive NP/OP swabs. In 2013, enrollment of healthy controls was initiated as part of ARI surveillance, which will provide data on proportional etiology of these pathogens in this population.

Our data demonstrated a markedly higher incidence of hospitalized ARI in rural population. Some locations have demonstrated lower incidence rates of respiratory infections in rural populations due to increased distance from hospital facilities.^35^, ^36^ Distance and associated travel time is not a barrier to hospital access in this population, as 96% of respondents in the Damanhour Healthcare Utilization Survey reported travel time to one of the surveillance facilities as less than one hour.^9^ Higher incidence rates in the rural locality were also demonstrated in Guatemala,^21^ possibly indicating that another aspect of rural residence is associated with higher rates of disease and/or hospitalization.

One potential explanation for the higher rate of hospitalization in the rural population is that admitting physicians are more likely to hospitalize rural patients presenting with even moderate or possibly progressive respiratory symptoms. Our data showed that a lower proportion of rural patients presented with severe symptoms and a higher percentage of admitted rural patients were under 5 years of age. It is possible that rural children were hospitalized as a precautionary measure in order to provide disease management that may be unavailable in their village and/or prevent progression to severe disease.

Our results are subject to several limitations. Infants <31 days were not enrolled; therefore, we do not have data on the epidemiology of viral pathogens in this age group and it is possible that we have underestimated the incidence of hospitalized ARIs in children aged 1 month <5 years. However, published estimates on neonates aged <1 month demonstrated proportional viral etiologies similar to patients aged 31 days to <1 year in our study.^37^ Additionally, although every effort was made to include all hospitalized infectious respiratory cases at our study sites, the inclusion criteria were biased toward certain symptomologies. We excluded patients aged 1 month to <5 years who presented without cough or other respiratory symptoms. However, several studies have since demonstrated that children in this age group with influenza infection may present in the absence of respiratory symptoms.^38^, ^39^

A larger‐than‐expected proportion of patients had a normal chest X‐ray despite a diagnosis of ARI. Chest radiography is a subjective measurement of disease and is dependent on individual physicians’ interpretations. Chest X‐rays in this study were interpreted by several physicians, who may have used different criteria to evaluate the results. Recent research has demonstrated that chest X‐rays may produce a higher rate of false negatives than previously thought,^40^ and patients may present with normal X‐ray results and develop infiltrates after admission.^41^

Two notable disruptions to study enrollment may have affected the seasonality data presented in this paper. Study activities were suspended for a one‐month period between November 1, 2009, and December 13, 2009, while the Egyptian Ministry of Health and Population diverted resources to developing a response to the 2009 H1N1 pandemic. Enrollment was suspended again on January 23, 2011, due to civil unrest during the 2011 Egyptian Revolution. Figure 2 clearly demonstrates decreased enrollment between January and April 2011 compared with other years. Although enrollment resumed on February 6, 2011, it is likely that the ongoing political unrest and climate of uncertainty affected healthcare utilization, operation of the public health system, or both. This also likely affected the estimated incidence rates for hospitalized ARI, which are markedly lower in 2011 than for other years. Despite the suspension of surveillance activities, seasonality was well defined for RSV, influenza A, and PIV‐3 and consistent with Northern Hemisphere seasonality of these pathogens.^42^, ^43^, ^44^, ^45^ Very few blood culture specimens from children aged 1 month to <5 years demonstrated positivity for any pathogen associated with ARI. The majority of positive specimens grew bacterial species more commonly found in normal skin flora, and likely indicates contamination during specimen collection. Additionally, challenges with sterile sample collection and the near universal use of antibiotics by our study population prior to hospitalization likely affected the detection rate of bacterial pathogens.^46^, ^47^ The accessibility and inappropriate use of antibiotics in Egypt is well documented^48^, ^49^; therefore, future studies on bacterial pathogens may require additional serology and PCR detection.

Previous data have demonstrated that adult ARI and pneumonia may be less commonly due to viral etiologies^50^ and our data seem to support this. However, the diagnostic panels used for this study did not include all common and possible causes of severe acute respiratory infection. Additional diagnostic methodologies on older children and adults, including blood cultures and sputum testing, may have yielded additional pathogens. The population‐based surveillance site in Damanhour is currently participating in a multisite study to determine the etiology of community‐acquired pneumonia. The study will test NP/OP swabs for an additional 19 bacterial and viral pathogens and provide a more complete picture of the epidemiology of ARI in the study population. Healthy controls will also be tested to determine proportional etiology for pathogens which may have some degree of baseline carriage or previous exposure in the study population.

## 5 Conclusion

This paper presents the first population‐based incidence rates of hospitalized ARIs in Egypt, along with data on the proportional contribution of several viral pathogens to the total burden of disease in each age group for the years reported. This data demonstrate the large burden of ARI hospitalizations in children aged 1 month to <5 years and the high proportional etiology of RSV in this age group. This study also highlights the need for additional methods of surveillance of bacterial and other viral pathogens in this population, which may reveal a substantial proportion of as yet unidentified etiologies in older adults.

## Declaration

The views expressed in this article are those of the author and do not necessarily reflect the official policy or position of the Department of the Navy, Department of Defense, nor the U.S. Government. The findings and conclusions in this report are those of the authors and do not necessarily reflect the official position of the Centers of Disease Control and Prevention. I am a military service member (or employee of the U.S. Government). This work was prepared as part of my official duties. Title 17 U.S.C. §105 provides that “Copyright protection under this title is not available for any work of the United States Government.” Title 17 U.S.C. §101 defines a U.S. Government work as a work prepared by a military service member or employee of the U.S. Government as part of that person's official duties.
